# Comparative Study of Octavinyl Oligomeric Sesquisiloxane Nanomaterial-Modified Asphalt Using Molecular Dynamics Method

**DOI:** 10.3390/polym14214577

**Published:** 2022-10-28

**Authors:** Lei Feng, Peng Zhao, Tongdan Chen, Minghai Jing

**Affiliations:** School of Materials Science and Engineering, Chang’an University, Xi’an 710061, China

**Keywords:** nano-OvPOSS, nano-ZnO, nano-SiO_2_, compatibility, modified asphalt

## Abstract

This paper mainly studies the compatibility and properties of octavinyl oligomeric silsesquioxane nanomaterial (nano-OvPOSS)-modified asphalt, in comparison with those of traditional zinc oxide nanomaterial (nano-ZnO) and silica nanomaterial (nano-SiO_2_), through the method of molecular dynamics simulation. Nano-OvPOSS, an organic–inorganic nano-hybrid material, is studied for the first time in the application of asphalt modification. By studying different sizes and types of nanomaterials, this paper elucidates the superiority of nano-OvPOSS as an asphalt modifier owing to the unique microstructure of eight organic groups of its inorganic framework. According to the results, nano-OvPOSS does not aggregate in the modified asphalt system and displays the best compatibility with asphalt when compared with nano-SiO_2_ and nano-ZnO. Moreover, nano-OvPOSS exhibits the most favorable compatibility with resinous oil out of the four asphalt components. The size of nano-OvPOSS determines its compatibility with asphalt. The smaller the particle size of nano-OvPOSS, the better its compatibility with asphalt. Therefore, out of all the four sizes of nano-OvPOSS (4.4 Å, 7 Å, 10 Å, and 20 Å) adopted in this study, the 4.4 Å nano-OvPOSS exhibits the best compatibility with asphalt. Additionally, compared with nano-SiO_2_ and nano-ZnO, nano-OvPOSS is capable of attracting more asphalt molecules around it so that it reduces the largest amount of ratio of free volume (RFV) of matrix asphalt, which can be reduced by 9.4%. Besides these characteristics, the addition of nano-OvPOSS into the matrix asphalt contributes to higher heat capacity, bulk modulus, and shear modulus of the asphalt system, which were increased by 14.3%, 74.7%, and 80.2%, respectively, thereby guaranteeing a more desirable temperature stability and deformation resistance in the asphalt system. Accordingly, nano-OvPOSS can be employed as a viable asphalt modifier to ensure a well-rounded performance of modified asphalt.

## 1. Introduction

Having been widely applied in the fields of industry, transportation, and infrastructure, asphalt is considered to be an important engineering material [1,2,3]. The current requirements for its capacity to withstand harsher conditions make it necessary to develop more modified asphalt with improved properties. Various studies about materials ranging from polymer-asphalt to polymer composites modified asphalt [4,5,6,7] and then to nanomaterial-modified asphalt have been conducted successively [8,9,10,11]. Recent years have constantly witnessed the universal addition of nanomaterial, such as zinc oxide nanomaterial (nano-ZnO) and silica nanomaterial (nano-SiO_2_) [12,13,14], into asphalt in order to improve polymer properties such as thermal stability, mechanical properties, machinability, and aging resistance [15,16,17].

What is worth mentioning is that there still leaves something to be desired about the usage of nanomaterials as asphalt modifiers. The performance of nanomaterial-modified asphalt is often influenced by the dispersion of nanomaterial in the asphalt. Since substances are always developing in the direction of energy reduction, a nanomaterial, which has small particle size and large surface energy, cannot maintain a constant dispersion state in asphalt for a long time and will undergo aggregation behavior. This will drastically minimize its advantages of nanometer effect [18]. Therefore, the nanomaterial-modified asphalt, because of the aggregation behavior, will gradually change its microstructure during storage, transportation, and service, thereby impairing the performance of pavements paved by the nanomaterial-modified asphalt.

To remove the aggregation of nanomaterial in asphalt, the pretreated methods, including mechanical dispersion, ultrasonic dispersion, and chemical surface modification, have been widely employed. In 2012, Khattak et al. [19] studied the impact of carbon nano-fiber modification on asphalt binder rheology. In order to solve the problem of nanomaterial enrichment in asphalt, the dispersion operations of sonication and high shear mixing were conducted. However, these treatments lead to such adverse consequences as nanomaterial damage and size damage, thus reducing the original performance of the asphalt binder. In 2020, Zhang et al. [20,21] estimated the compatibility between nanomaterial and asphalt. They found that, after organic modification, the benzyl chain in ODBA (octadecyl dimethyl benzyl ammonium chloride) increases the adsorption effect of asphalt molecules on individual REC (rectorite) layers, thereby contributing to the desired distribution of ODBA-REC in the asphalt system. However, it takes a long time to conduct this modification, since the preparation of ODBA-REC requires at least five standing hours so that the intercalation agents can fully enter the REC. Moreover, the treatment only realizes the entrance of the intercalation agent into the REC layer, yet the intercalation agent is not chemically combined with the REC. Shen and He et al. [22,23] prepared solvent-free calcium carbonate (CaCO_3_) nanofluids and solvent-free SiO_2_ nanofluids to solve the aggregation phenomena of nanoparticle in asphalt. They found that after the surface functionalization, the compatibility of nanomaterial with asphalt was improved, and the nanomaterials were evenly dispersed in asphalt. However, as is mentioned in their study, the addition of solvent-free nanofluids weakens the high-temperature rutting resistance of asphalt. Besides, this surface functionalization process is too complicated for use in a real operation.

Regardless of the complex process, the aggregation more often than not still could not be completely eliminated. To offset such a deficiency, POSS (polyhedral oligomeric silsesquioxane), a kind of organic–inorganic nanohybrid material possessing both the properties of organic and inorganic materials [24,25,26], was explored as an asphalt modifier in this study. In so doing, we made an attempt to avoid the occurrence of the above-mentioned unfavorable situations, and at the same time better the performance of modified asphalt on the whole. POSS is easily functionalized by changing the organic groups; this, along with its potential to undergo grafting reactions or copolymerization, enabled to have better compatibility with asphalt as well as superb convenience to be incorporated into polymer through blending. POSS was first synthesized in 1946 by Scott [27]. Afterwards, many kinds of POSS were prepared and utilized, such as OvPOSS (octavinyl oligomeric silsesquioxane), CyPOSS (cyclohentyl POSS), and CpPOSS (cyclopentyl POSS) [28,29,30,31,32,33]. In 2009, Yu et al. [34] prepared nylon composites by melt-blending OvPOSS and Ocs-POSS (octaepoxycyclohexyl dimethyl silyl POSS) and found that these nylon composites exhibited higher decomposition temperatures. In 2010, Xu and Yang et al. [35,36] synthesized and studied PAS-POSS (poly acetoxystyrene OvPOSS) and PS-POSS (poly styrene OvPOSS) and found that the incorporation of POSS into acetoxystyrene or styrene can improve the thermal properties of original polymeric material through the measurements of DSC and TGA. In 2022, Hao et al. [37] found that after adding POSS, the tensile strength and tensile modulus of the epoxy polymer were enhanced by 22.9% and 31.6%. It can be concluded from these experiments that POSS, as an excellent polymer copolymer, is capable of improving the oxidation resistance, mechanical property, and aging resistance of polymer.

In this paper, nano-OvPOSS was selected to be the study object because nano-OvPOSS contains eight vinyl groups which are common in polymers. Such structural similarity smooths the reciprocal effect between nano-OvPOSS and polymer, and therefore helps to obtain to the targeted result. Meanwhile, through the comparison of nano-OvPOSS with traditionally used nano-ZnO and nano-SiO_2_, this study aims to analyze the superiority of nano-OvPOSS in its compatibility with matrix asphalt as well as its effects on the properties of matrix asphalt. Using the method of molecular dynamic simulation, which is based on Newton’s law and predicts the macroscopic performance of materials by calculating the intra- and intermolecular interactions, the structure–property relationship of the organic–inorganic composite material was studied and the radial distribution function (RDF), density, mixing energy, aggregation behavior, ratio of free volume (RFV), heat capacity (C_v_), and mechanical properties of the asphalt system were calculated and analyzed. This paper conducted an exploration into the feasibility of nano-OvPOSS to be used as an asphalt modifier, which is here studied for the first time in the field of asphalt modification, with the intention of providing a new direction to investigate more efficient asphalt modifiers.

## 2. Simulation Models and Methods

### 2.1. Molecular Models of Asphalt

**Figure 1:**
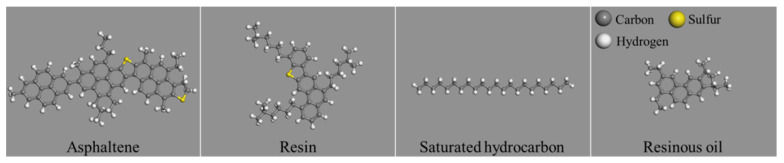
Molecular structure of four asphalt components.

The four components of the model of asphalt used by Hansen [38], based on the results of extensive experiments, were adopted in this study, including asphaltene, resin, saturated hydrocarbon, and resinous oil, as is shown in Figure 1.

### 2.2. Molecular Models of Nanomaterials

**Figure 2:**
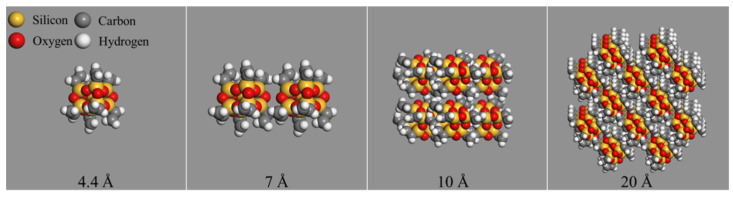
OvPOSS nanocluster with different sizes.

OvPOSS nanocluster models with different sizes (4.4 Å, 7 Å, 10 Å, and 20 Å) were constructed, as is shown in Figure 2. The nano-OvPOSS with eight organic branches on its framework can display its structural superiority in interacting with asphalt. The sphere diameters of nanoclusters are referred to as the size of nanoclusters in this study. The 4.4 Å OvPOSS nanocluster, composed of only one molecule, was chosen to construct the nanomaterial-modified asphalt model because this particle size was the smallest size that can be constructed by the nanocluster building tool of simulation software.

**Figure 3:**
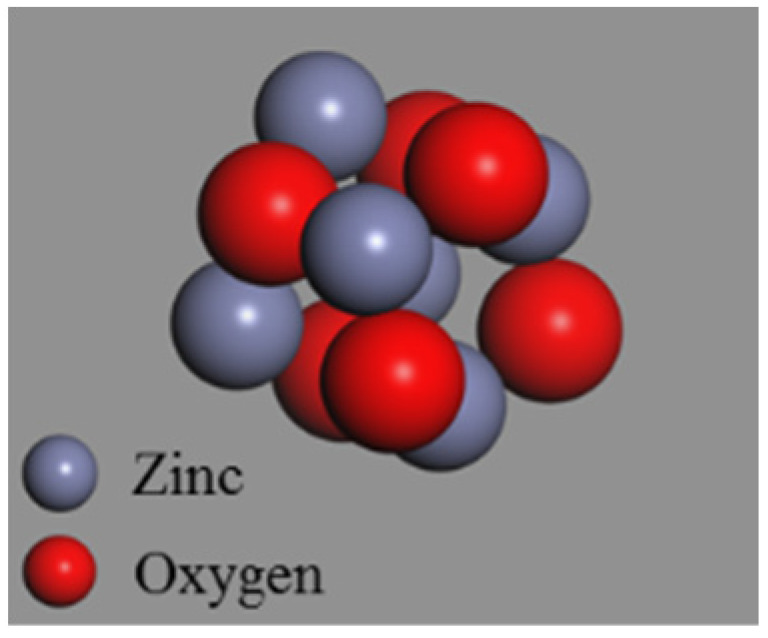
ZnO nanocluster.

**Figure 4:**
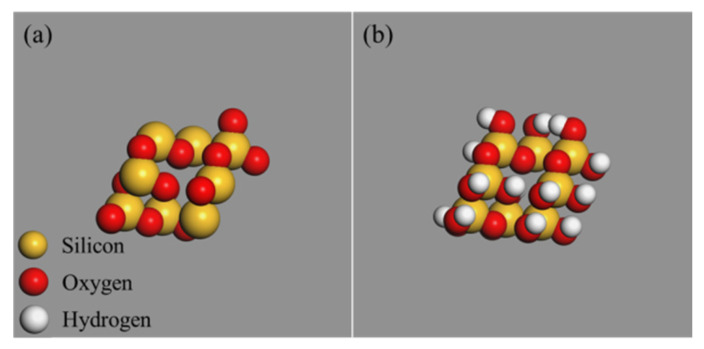
SiO_2_ nanocluster: (**a**) original SiO_2_ nanocluster; (**b**) modified SiO_2_ nanocluster.

For comparative analysis, the 4.4 Å ZnO nanocluster and 4.4 Å SiO_2_ nanocluster were also constructed by the nanocluster building tools of simulation software, as control groups, to be consistent with the particle size of OvPOSS nanocluster. To eliminate the unsaturated boundary effect of SiO_2_ nanocluster, hydrogen atoms were added to the unsaturated oxygen atoms and hydroxyl groups were added to the unsaturated silicon atoms on the surface of SiO_2_ nanocluster. Figure 3 shows the molecular structure of ZnO nanocluster. Figure 4 shows the molecular structure of original SiO_2_ nanocluster and modified SiO_2_ nanocluster.

### 2.3. Construction of the Modified Asphalt Models

**Figure 5:**
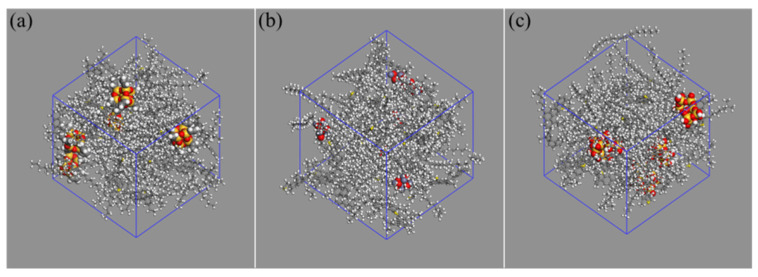
Nanomaterial-modified asphalt models: (**a**) nano-OvPOSS-modified asphalt; (**b**) nano-ZnO-modified asphalt; (**c**) nano-SiO_2_-modified asphalt.

The construction of nanomaterial-modified asphalt models was based on the four-component asphalt model and nanomaterial models. The number of nanoclusters in nano-OvPOSS-modified asphalt models was chosen to be 5, since it was the least amount required in a simulation study to obtain adequately representative data displaying the kinetic and thermodynamic properties, especially the aggregation behavior of nanomaterials. In that case, the content of 5 OvPOSS nanoclusters in modified asphalt was determined to be 11% through simulation. To ensure a distinct simulation comparison between nano-OvPOSS, nano-ZnO, and nano-SiO_2_, the contents of nano-ZnO and nano-SiO_2_ were set to be 11%, respectively, the same as that of nano-OvPOSS. In this case, the number of ZnO nanoclusters was found to be 6 and that of SiO_2_ nanoclusters to be 5 through the corresponding simulation. Therefore, in this molecular simulation study, the content of 11% is appropriate to manifest the difference of the effects of nano-OvPOSS, nano-ZnO, and nano-SiO_2_ on the properties of asphalt from the microscopic perspective. The molecular formula and total number of molecules contained in the three nanomaterial-modified asphalt systems are shown in Table 1. The initial models are presented in Figure 5.

### 2.4. Simulation Process

**Figure 6:**
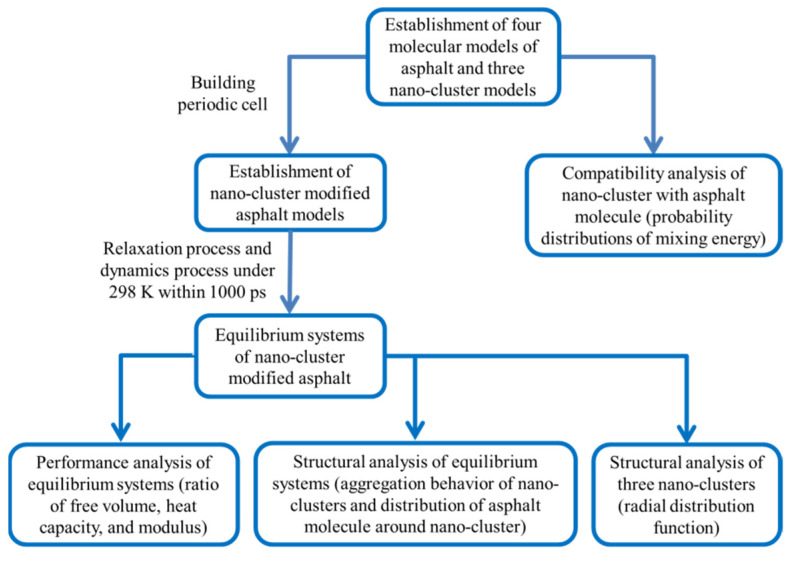
Flow chart of simulation study.

The Amorphous Cell tool of Materials Studio (MS) was adopted to construct the modified asphalt models with three-dimensional periodic cell at room temperature. Additionally, then the Forcite tool and Blends tool were employed to perform the dynamics and compatibility simulation. The initial density of model was set as 1.0 g/cm^3^ and the time step was 1.0 fs. The COMPASS (condensed-phase-optimized molecular potential for atomistic simulation studies) force field [39,40] was used to characterize the atomistic interactions. The temperature and pressure in the molecular dynamics simulation process were controlled by the Nose [41] thermostat and Berendsen [42] barostat, respectively. Additionally, the decay constant of the temperature control method and pressure control method were both 0.1 ps. Van der Waals interactions and electrostatic interactions were calculated by an atom-based method with a cut off distance of 12.5 Å and PPPM (particle–particle–particle mesh) method with a cut off distance of 12 Å, respectively. Firstly, the geometry optimization program was used to optimize the structure of the initial model, and then the dynamic process for 4 ns was employed to ensure a stable asphalt molecular configuration. Secondly, to obtain the true global potential energy minimum configuration, the anneal process was used to overcome the migration energy barrier in the asphalt system. In this process, the prepared asphalt model was initially heated up to 500 degrees Kelvin (K), and then cooled to 298 K. This anneal process was carried out four times for a total of 4 ns. Thirdly, after these relaxation procedures, the geometry optimization program was used for a second time, and then the molecular dynamic process with the NPT ensemble for 1 ns (298 K, 1 atm) was followed. Additionally, the flow chart of simulation study is shown in Figure 6.

### 2.5. Molecular Dynamics Simulation Theories

#### 2.5.1. Structural Parameters

In this paper, RDF and RVF were used to describe the structure of models, and the details are shown in Table 2.

#### 2.5.2. Compatibility

Based on the Flory–Huggins theory, the expression of free energy of mixing of a mixed system is expressed by Equations (1) and (2):

(1) $$\frac{\Delta G}{RT}=\frac{\phi_{b}}{n_{b}}ln\phi_{b}+\frac{\phi_{s}}{n_{s}}ln\phi_{s}+x\phi_{b}\phi_{s}$$

(2) $$x=\frac{E_{\mathrm{mix}}}{k_{B}T}$$

where *R* is the gas constant; *k_B_* is the Boltzmann constant; *T* is the test temperature; Δ*G* is the free energy of mixing; E_mix_ is the mixing energy; *ϕ_i_* is the volume fraction of *i*; *n_i_* is the degree of polymerization of *i*; *x* is the interaction parameter.

In the Flory–Huggins model, each component occupies a lattice site. For a lattice with coordination number *Z*, the mixing energy can be calculated by Equation (3):

(3) $$E_{\mathrm{mix}}=\frac{1}{2}Z\left(E_{\mathrm{bs}}+E_{\mathrm{sb}}-E_{\mathrm{bb}}-E_{\mathrm{ss}}\right)$$

where *E_ij_* is the binding energy between component *i* and component *j*.

## 3. Results and Discussion

### 3.1. Basic Information

In this section, the structural characteristics of the three nanomaterials were analyzed through RDF, and the accuracy of the simulation model and simulation method was verified through the curve of density and total energy as a function of simulation time.

#### 3.1.1. The RDF of the Three Nanomaterials

**Figure 7:**
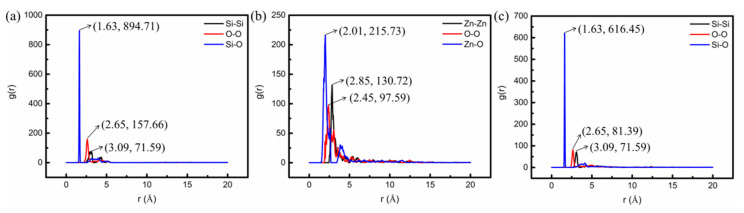
RDF of the host atoms of the three nanoclusters: (**a**) OvPOSS; (**b**) ZnO; (**c**) SiO_2_.

To study the difference of molecular structures of the three nanomaterials involved in this paper, and to clarify the structural superiority of nano-OvPOSS, the RDF of the host atoms (Si, Zn, and O) of the three nanomaterials in the asphalt simulation systems was studied. RDF is a measure to characterize the packing state of atoms and the distance between atoms. The simulation results are shown in Figure 7. For nano-OvPOSS and nano-SiO_2_, silicon atoms and oxygen atoms were selected as the reference and selection, respectively; for nano-ZnO, zinc atoms and oxygen atoms were selected as the reference and selection, respectively.

According to Figure 7, the curves of RDF of nano-OvPOSS and nano-SiO_2_ both have the first peak at 1.63 Å, representing the distance between silicon atoms and oxygen atoms; the second peak of the curve of RDF is at 2.65 Å, representing the distance between oxygen atoms; the third peak of the curve of RDF is at 3.09 Å, representing the distance between silicon atoms. Meanwhile, the curve of RDF of nano-ZnO has the first peak at 2.01 Å, representing the distance between zinc atom and oxygen atom; the second peak of the curve of RDF is at 2.45 Å, representing the distance between oxygen atoms; the third peak of the curve of RDF is at 2.85 Å, representing the distance between zinc atoms. Additionally, the intensity of the first peak, second peak, and third peak of nano-OvPOSS is 894.71%, 157.66%, and 71.59%; the intensity of the first peak, second peak, and third peak of nano-ZnO is 215.73%, 97.59%, and 130.72%; the intensity of the first peak, second peak, and third peak of nano-SiO_2_ is 616.45%, 81.39%, and 71.59%.

According to the above statistics, conclusions can be drawn that the host atoms of nano-ZnO are totally different in distance as well as in packing density from those of nano-SiO_2_ and those of nano-OvPOSS. Yet, the host atoms of nano-OvPOSS are nearly the same as those of nano-SiO_2_ in the distance. However, the packing density of Si-O and O-O of nano-OvPOSS is particularly stronger than that of Si-O and O-O of nano-SiO_2_. Such distinctive strength of packing density of the host atoms of nano-OvPOSS is owing to the influence of its eight organic functional groups.

Despite the fact that the host atoms of nano-OvPOSS and nano-SiO_2_ are the same, which are silicon and oxygen, the structure of nano-OvPOSS, an organic–inorganic nano-hybrid material, is apparently differentiated from nano-SiO_2_, an inorganic nanomaterial. It is this structural difference from nano-SiO_2_ and nano-ZnO that gives nano-OvPOSS the better performance when used as an asphalt modifier. As for nano-SiO_2_, since it cannot react with asphalt molecules, its main function in asphalt is attributed to its nanoparticle structure. Nano-OvPOSS, by contrast, not only reacts with asphalt molecules, but also functions as a nanoparticle, because it perfectly bears both the characteristics of polymers and inorganic nanoparticles.

#### 3.1.2. Model Validation

**Figure 8:**
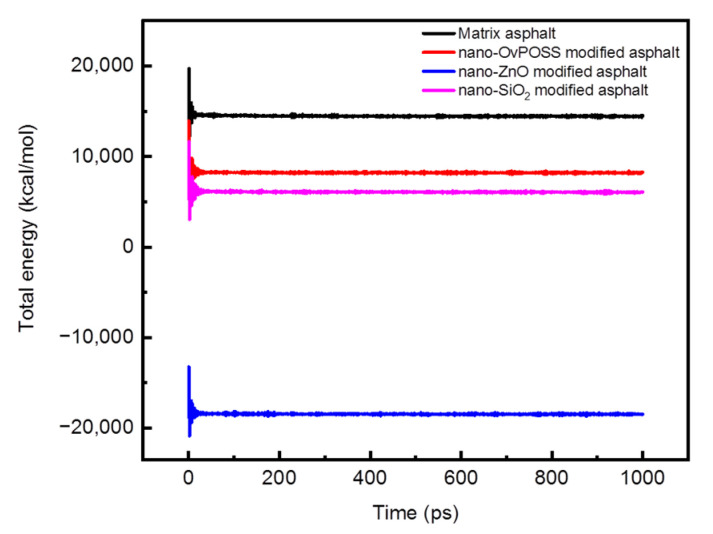
Total energy of asphalt systems.

**Figure 9:**
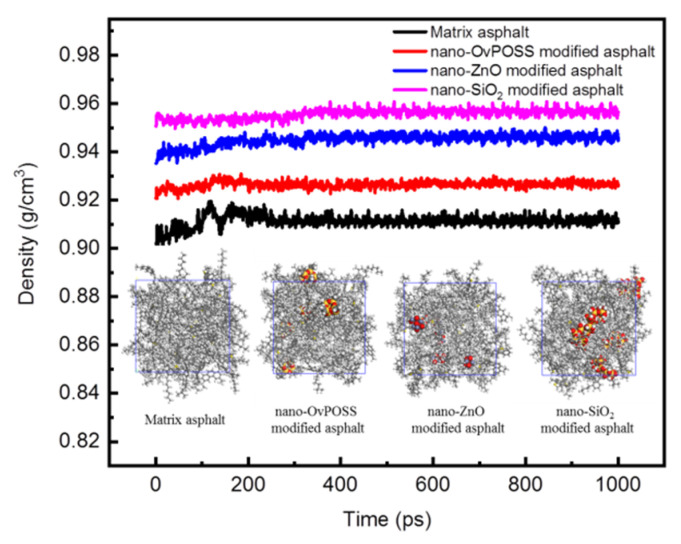
Density of asphalt systems.

The total energy and density of four asphalt simulation systems as a function of simulation time are shown in Figure 8 and Figure 9. As can be seen from Figure 8 and Figure 9, the separate addition of these three nanomaterials all can decrease the total energy of the matrix asphalt system and increase the density of the matrix asphalt. Another point which can be drawn from Figure 8 and Figure 9 is that the total energy and the density of various simulated system basically remain unchanged during the dynamic process of 1000 ps, which means the relaxation process before this dynamic process has brought the simulated systems to a substantially equilibrized state. The dynamic results of the last 600 ps were chosen as the data reference for subsequent calculation, during which time the most stable state for a simulation system has been attained.

### 3.2. Compatibility Analysis

The eight vinyl groups of nano-OvPOSS endow this nanomaterial with the distinctive potential to be compatible with polymers. This can be supported by the molecular dynamics method that simulates the probability distribution of the mixing energy of four asphalt components with nano-OvPOSS, nano-ZnO, and nano-SiO_2_, respectively, and the probability distribution of the mixing energy between four asphalt components and nano-OvPOSS at different sizes (4.4 Å, 7 Å, 10 Å, and 20 Å). The compatibility analysis is based on the Flory–Huggins model, which is the most famous theory of the thermodynamics of mixing in binary systems [43,44,45,46]. Additionally, the mixing energy characterizing the compatibility of the two materials was calculated by the Blends tools of MS in this paper.

#### 3.2.1. The Mixing Energy of Asphalt Molecules with Nano-OvPOSS, Nano-ZnO, and Nano-SiO_2_, Respectively

**Figure 10:**
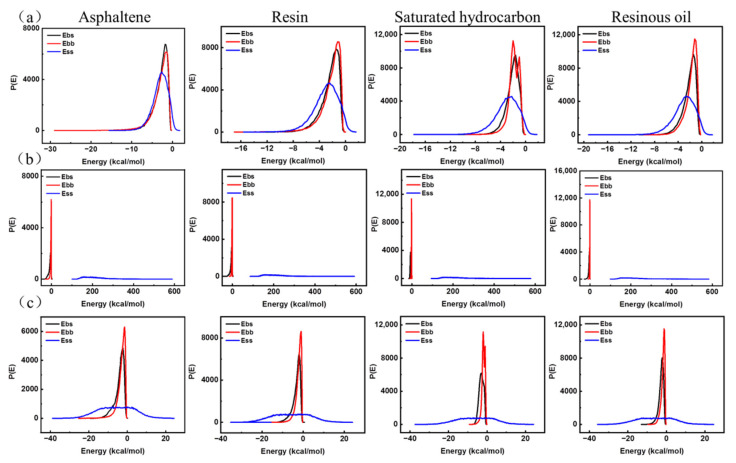
The probability distribution of mixing energy of nanomaterials with four asphalt components: (**a**) nano-OvPOSS with four asphalt components; (**b**) nano-ZnO with four asphalt components; (**c**) nano-SiO_2_ with four asphalt components.

The nanomaterials were used as the screen, and the components of asphalt were used as the base. The probability distributions of mixing the energy of four asphalt components with nano-OvPOSS, nano-ZnO, and nano-SiO_2_ (10 Å) were calculated, respectively, using the Blends tool, as is shown in Figure 10.

In Figure 10a, it can be seen that the curves of E_bb_ (base–base), E_bs_ (base–screen), and E_ss_ (screen–screen) of nano-OvPOSS with asphaltene, resin, saturated hydrocarbon, and resinous oil are basically similar to each other. In contrast, the curves of E_bb_, E_bs_, and E_ss_ of nano-ZnO with asphaltene, resin, saturated hydrocarbon, and resinous oil are obviously different from each other in Figure 10b; in Figure 10c, the curves of E_bb_, E_bs_, and E_ss_ of nano-SiO_2_ with asphaltene, resin, saturated hydrocarbon, and resinous oil are also different from each other. Therefore, the curves of nano-OvPOSS with four asphalt components show the most distinguishable similarity when compared with those of nano-ZnO, and those of nano-SiO_2_ with four asphalt components. It is generally accepted that if two materials exhibit similar curves of E_bb_, E_bs_, and E_ss_, the compatibility of the two materials is extremely satisfying. As a result, the mixing energy of asphalt molecules with respective nano-OvPOSS, nano-ZnO, and nano-SiO_2_ proves the fact that nano-OvPOSS outperforms nano-ZnO and nano-SiO_2_ in its compatibility with four asphalt components.

**Figure 11:**
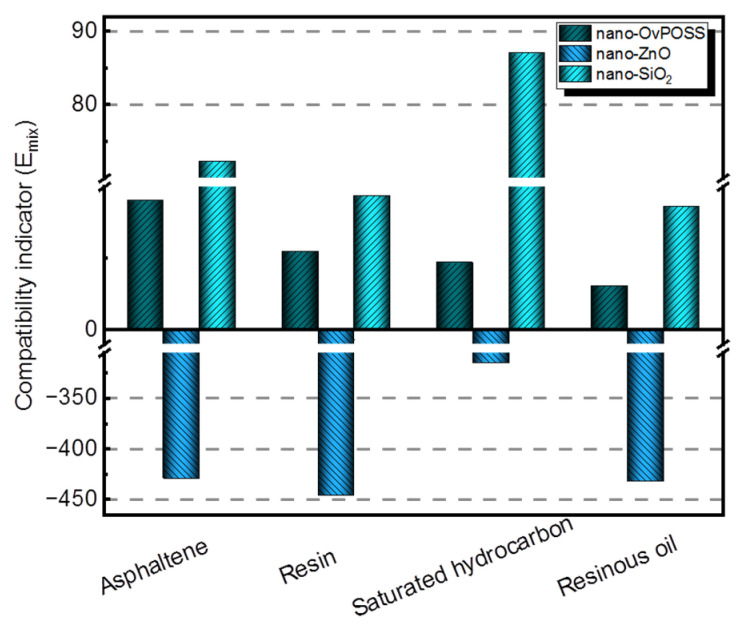
Compatibility indicator E_mix_ of nanomaterials with four asphalt components.

To be more specific, the quantitative analysis is also studied. Figure 11 expresses the compatibility indicator E_mix_, which is concerned with the quantitative analysis of the compatibility of the three nanomaterials with four asphalt components. If the absolute value of E_mix_ is the smallest, the compatibility of two materials is the best. It can be seen from Figure 11 that the absolute values of E_mix_ of nano-OvPOSS with four asphalt components are the lowest, all of which are below 70. In addition, it becomes apparent that the absolute values of E_mix_ of nano-ZnO with corresponding asphalt components are the highest, which are all above 300; it is further determine that the absolute values of E_mix_ of nano-SiO_2_ with corresponding asphalt components lie in between. The simulation results present the compatibility order of the three nanomaterials with asphalt components: that is to say, nano-OvPOSS offers the best compatibility with asphalt, followed by nano-SiO_2_ and nano-ZnO subsequently.

Since there exists a conspicuous difference in the structure and properties between metal atoms and non-metal atoms, it is obvious that the metallic oxide ZnO is almost incompatible with asphalt components. As for nano-SiO_2_, despite its similar framework structure of silicon and oxygen to nano-OvPOSS, it is actually overshadowed by nano-OvPOSS when it comes to compatibility with asphalt components. Basically, the structural equivalence guarantees the compatibility of different materials. It is the eight organic groups of nano-OvPOSS interacting with the active groups of asphalt molecule that endows nano-OvPOSS with superb compatibility with asphalt. Yet, nano-SiO_2_ and nano-ZnO, with no organic groups, do not show such a performance.

#### 3.2.2. The Mixing Energy between Four Asphalt Components and Nano-OvPOSS with Different Sizes

**Figure 12:**
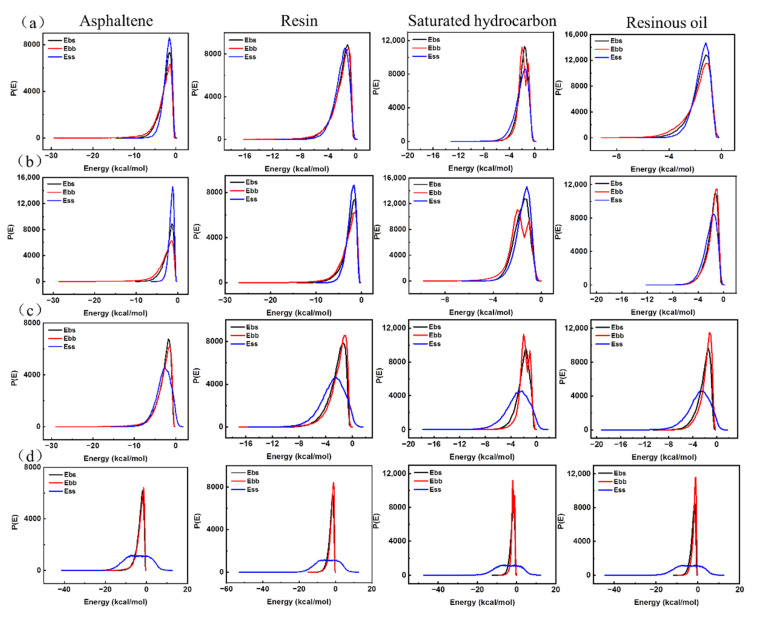
The probability distribution of mixing energy of nano-OvPOSS with asphaltene, resin, saturated hydrocarbon, and resinous oil: (**a**) 4.4 Å nano-OvPOSS; (**b**) 7 Å nano-OvPOSS; (**c**) 10 Å nano-OvPOSS; (**d**) 20 Å nano-OvPOSS.

The compatibility between four asphalt components and nano-OvPOSS with different sizes was further investigated to figure out the best particle size of nano-OvPOSS that is compatible with asphalt. The nano-OvPOSS was used as the screen, and the asphalt components were used as the base. The probability distributions of mixing energy of four asphalt components with nano-OvPOSS (4.4 Å, 7 Å, 10 Å, and 20 Å) were calculated, respectively, using Blends tool, as is shown in Figure 12.

Figure 12a shows the probability distribution of mixing energy of 4.4 Å nano-OvPOSS with asphaltene, resin, saturated hydrocarbon, and resinous oil; Figure 12b–d show the probability distribution of mixing energy of 7 Å, 10 Å, and 20 Å nano-OvPOSS with four asphalt components. It can be seen from Figure 12a that the probability distribution curves of E_bb_, E_bs_, and E_ss_ in these four pictures are very similar to each other. However, it can be seen from Figure 12b–d that, with the increase in nano-OvPOSS particle size, the probability distribution of E_bb_, E_bs_, and E_ss_ of nano-OvPOSS with four asphalt components gradually changed from similar to different. Conclusions can be drawn, namely that the size of nano-OvPOSS plays a decisive role in its compatibility with asphalt components. Generally, the smaller the particle size of nano-OvPOSS, the better its compatibility with asphalt components. Of note is that nano-OvPOSS with a particle size of 4.4 Å has the best compatibility with asphalt components.

**Figure 13:**
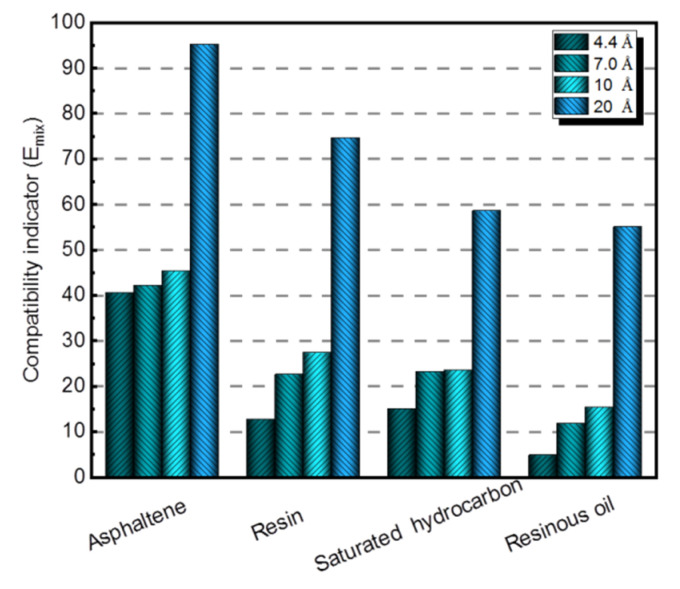
Compatibility indicator E_mix_ of nano-OvPOSS in different sizes with four asphalt components.

Figure 13 is about the quantitative analysis of the compatibility of nano-OvPOSS in different sizes with four asphalt components, which expresses the compatibility indicator E_mix_ of asphalt blends. As can be seen, the absolute value of E_mix_ of 4.4 Å nano-OvPOSS with each asphalt component is the smallest compared with that of 7 Å, 10 Å, and 20 Å nano-OvPOSS. With the increase in the particle size of nano-OvPOSS, the absolute value of E_mix_ of nano-OvPOSS with four asphalt components also increases. Simultaneously, compared with the other three asphalt components, the resinous oil expresses the smallest absolute value of E_mix_ of each size of nano-OvPOSS. Conclusions can be drawn that the four sizes of nano-OvPOSS all have the best compatibility with resinous oil, and that the smaller the particle size of nano-OvPOSS is, the better its compatibility with asphalt components will be.

It is generally accepted that nanomaterial exhibits a large specific surface area which is closely related to its uniquely small size [47,48,49]. For this study, the smaller the size of nano-OvPOSS, the larger the specific surface area, and thus the larger the contact range between asphalt molecules and organic groups on the surface of OvPOSS nanoparticles. As the interaction between nanomaterial and asphalt components becomes stronger, its compatibility with asphalt components becomes better [50], which explains why 4.4 Å nano-OvPOSS performs the best compatibility and was chosen as the study subject.

### 3.3. Distribution of Asphalt Molecule around Nanomaterial

**Figure 14:**
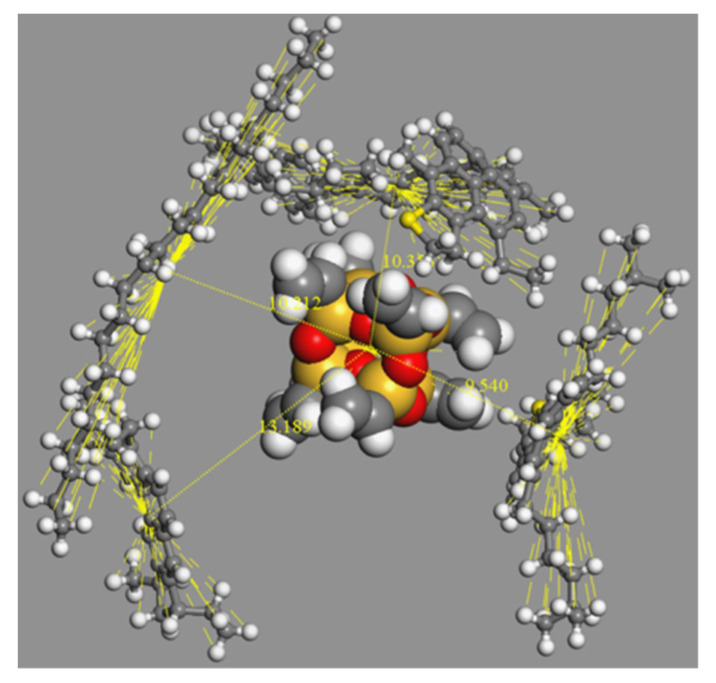
The distribution explanatory drawing of asphaltene, resin, saturated hydrocarbon, and resinous oil around OvPOSS nanocluster.

In this section, the distribution of asphalt molecules around nano-OvPOSS, nano-ZnO, and nano-SiO_2_ was analyzed to clarify the superiority of nano-OvPOSS as asphalt nano-modifier. The distance between the centroid of nanocluster and the centroid of asphalt molecule was taken as the distance between nanocluster and asphalt molecule. The cut off distance in the molecular dynamics process was set as 12.5 Å. With the inclusion of the distance between the centroid of nanocluster and its surface atoms which was about 2.5 Å, the cut off distance was therefore chosen to be 15 Å for data counting. Figure 14 shows the distribution explanatory drawing of asphaltene, resin, saturated hydrocarbon, and resinous oil around OvPOSS nanocluster. As is shown in the figure, the number of the asphaltene molecules around the OvPOSS nanocluster is 1, and the distance between the centroid of asphaltene molecule and the centroid of OvPOSS nanocluster is 10.4 Å; as for the resin molecule, the number is 1 and the distance is 9.5 Å; as for the saturated hydrocarbon molecule, the number is 1 and the distance is 10.3 Å; as for the resinous oil molecule, the number is 1 and the distance is 13.2 Å. In conclusion, the average distance between OvPOSS nanoclusters and asphalt molecules is 10.9 Å and the average number of asphalt molecules around the OvPOSS nanocluster is 1.

After performing an assessment of the types and numbers of asphalt molecules around a nanocluster, as well as calculating of the average distance between nanoclusters and asphalt molecules from the simulation results of the distribution map of asphalt molecules around nanocluster, the details of the distribution data are presented in Table 3, Table 4 and Table 5.

According to Table 3, Table 4 and Table 5, in the nano-OvPOSS-, nano-ZnO-, and nano-SiO_2_-modified asphalt system, respectively, the total amount of saturated hydrocarbon and of resinous oil around the nanomaterial is larger than that of asphaltene and that of resin. This can be explained by the fact that, in the asphalt colloid system, there is a large distribution of saturated hydrocarbon and resinous oil acting as a peptizer around asphaltene and resin.

As is shown in the above statistics, an average of 9.8 asphalt molecules distribute around one OvPOSS nanocluster, compared with an average of 7.6 asphalt molecules around one ZnO nanocluster, and 8.2 asphalt molecules around one SiO_2_ nanocluster. Meanwhile, the average distance between nano-OvPOSS and asphalt molecules is 11.1 Å, which is shorter than the average distance between nano-ZnO and asphalt molecules (14.2 Å), and shorter than the average distance between nano-SiO_2_ and asphalt molecules (12.1 Å). Conclusions can be drawn that nano-OvPOSS attracts more asphalt molecules around it than nano-ZnO or nano-SiO_2_ does, and at the same time nano-OvPOSS keeps closer average distance from asphalt molecules than nano-ZnO or nano-SiO_2_ does.

That nano-OvPOSS performs good absorption with asphalt molecules makes it more compatible with asphalt, thus helping to form more stable structure asphalt. This positive quality of nano-OvPOSS of absorbing more asphalt molecules is because the eight organophilic groups of nano-OvPOSS possess the ability to absorb and link asphalt molecules, which, however, is not achievable for nano-ZnO and nano-SiO_2_.

When blended with matrix asphalt, the nanomaterial will fill in the gaps between the asphalt molecules under the action of its own macro quantum tunnel and volume effect. After that, nanoparticles will combine with macromolecules in asphalt to form a stable three-dimensional network structure. The better the compatibility of the nanomaterial with asphalt, the more stable the three-dimensional network structure. Then, the cohesive energy, mechanical properties, and rheological properties of matrix asphalt will be correspondingly enhanced [51]. Therefore, the favorable compatibility of nano-OvPOSS with asphalt is also able to bring out more beneficial properties of matrix asphalt.

### 3.4. Properties Comparison

This section analyzed the aggregation behavior of nanoclusters in the equilibrium system, as well as the RFV, C_v_, and modulus of the equilibrium system, to clarify the feasibility of nano-OvPOSS as a new nano-modifier of asphalt.

#### 3.4.1. Aggregation Behavior

With high surface energy, the nanomaterials are easy to aggregate because substances always develop in the direction of energy reduction. Therefore, to investigate the aggregation behavior of the nanomaterials in the asphalt system, we studied the distribution of nano-OvPOSS, nano-ZnO, and nano-SiO_2_ in the asphalt system.

**Figure 15:**
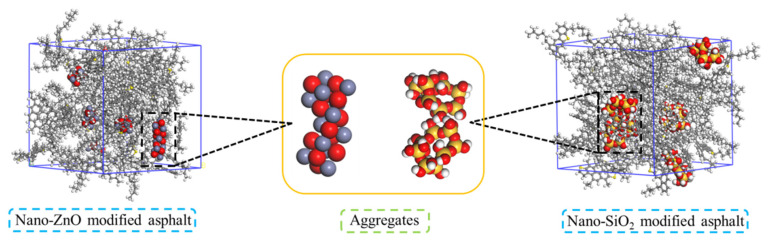
Aggregation behavior of nano-ZnO and nano-SiO_2_ in asphalt systems.

**Figure 16:**
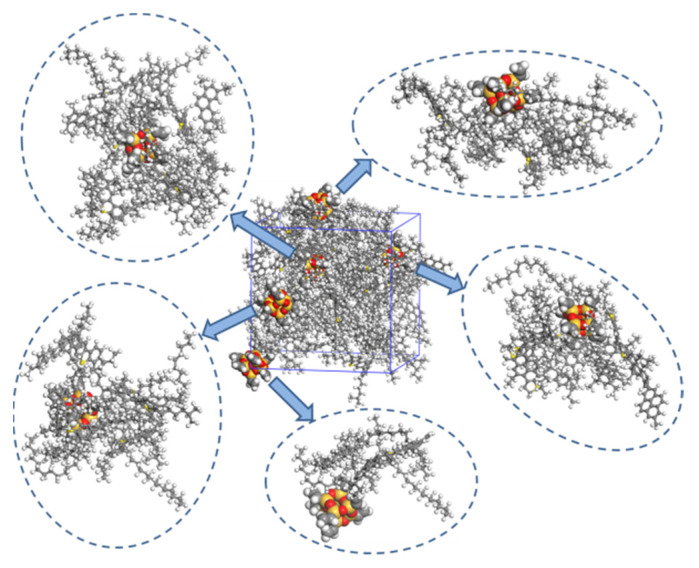
The distribution of nano-OvPOSS in the asphalt system.

Figure 15 and Figure 16 show the microstructure of the three modified asphalt systems. It can be seen from Figure 15 that nano-ZnO aggregates in the asphalt system, as does nano-SiO_2_. However, nano-OvPOSS, as is shown in Figure 16, evenly disperses in the asphalt system without aggregation. This is because nano-OvPOSS can produce physical and chemical adsorption with asphalt molecules thanks to the distribution of eight vinyl groups on the surface of the cage frame structure. Moreover, the cage-shaped skeleton structure of nano-OvPOSS provides a firm supporting effect, which enables nano-OvPOSS to combine with asphalt molecules to form a stable structural asphalt.

#### 3.4.2. The Free Volume of Asphalt Systems

**Figure 17:**
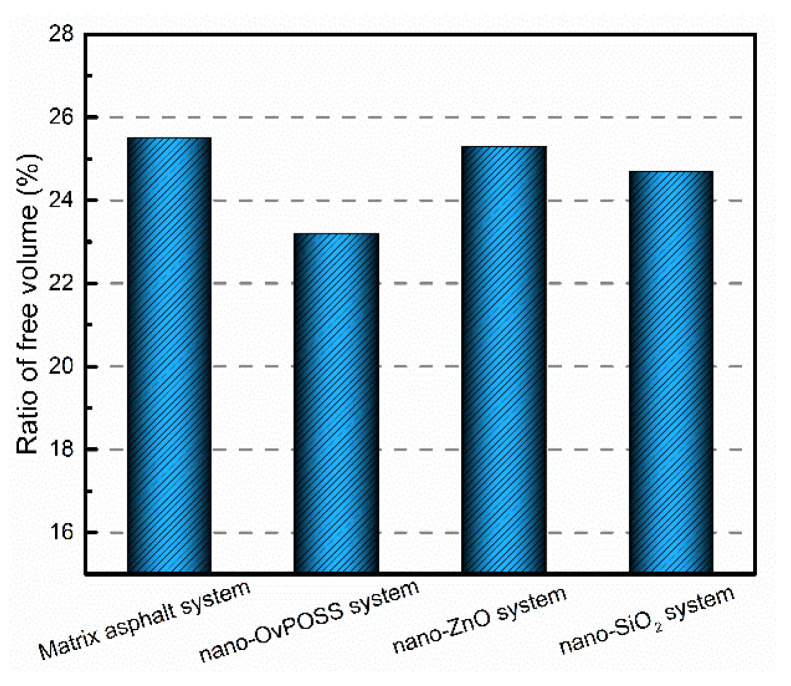
RFV of asphalt systems.

**Figure 18:**
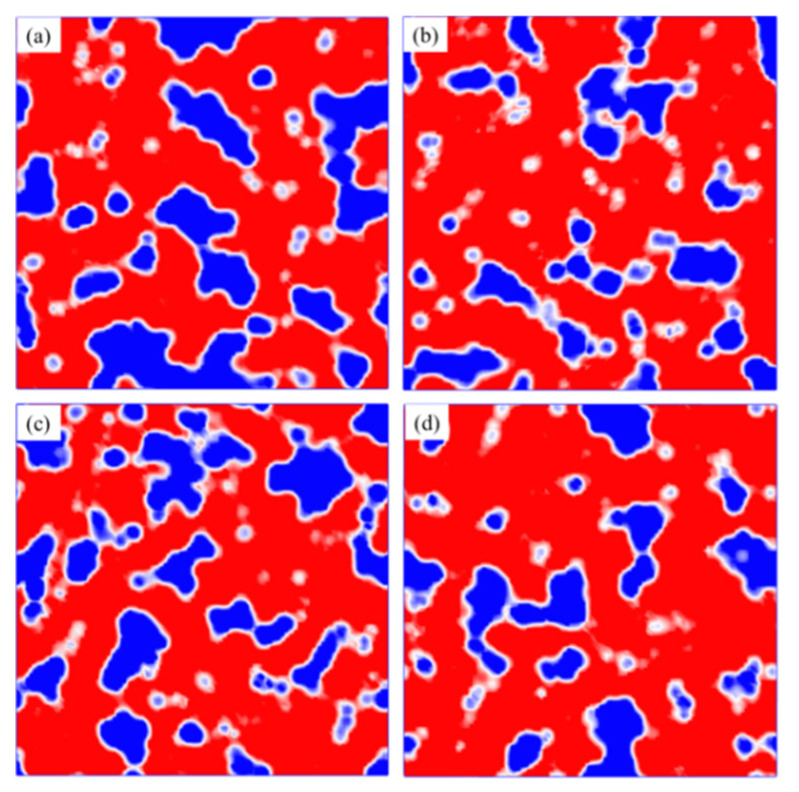
The free volume and occupied volume of asphalt systems: (**a**) matrix asphalt; (**b**) nano-OvPOSS-modified asphalt; (**c**) nano-ZnO-modified asphalt; (**d**) nano-SiO_2_-modified asphalt (Free volume is blue and occupied volume is red).

We used free volume theory to analyze the change in diffusivity and viscosity of polymer [52,53]. If the RFV of asphalt decreases, the fluidity of asphalt decreases and its viscosity increases, which consequentially improves the deformation resistance of asphalt. Figure 17 and Figure 18 present the changes in RFV and the free volume of matrix asphalt before and after the separate addition of nano-OvPOSS, nano-ZnO, and nano-SiO_2_ into the matrix asphalt.

As is shown in Figure 17, without the nanomaterials, the value of RFV is 25.5%. With the separate addition of nano-OvPOSS, nano-ZnO, and nano-SiO_2_, the value of RFV reduces to 23.1%, 25.3%, and 24.4%. It can be seen that the RFVs of matrix asphalt and the three nanomaterials-modified asphalt are all between 20–30%, and that the separate addition of nano-OvPOSS, nano-ZnO, and nano-SiO_2_ all leads to the shrinking of the free movement space of molecules in the asphalt system. Compared with nano-ZnO and nano-SiO_2_, nano-OvPOSS reduces the largest amount of RFV of matrix asphalt, which can be reduced by 9.4%. This is because its unique structure of eight vinyl groups allows nano-OvPOSS to reinforce its combination with asphalt molecules and promote a non-bonding interaction.

The effect of nano-OvPOSS on the RFV of matrix asphalt is be illustrated in Figure 18, where the blue area stands for the free volume. It can be seen that the blue area in Figure 18a is large and contiguous. However, after the addition of nano-OvPOSS, as is shown in Figure 18b, the blue area becomes smaller and less contiguous. Moreover, compared with Figure 18c of nano-ZnO and Figure 18d of nano-SiO_2_, the blue area of Figure 18b is the smallest and most dispersed. These changes in the blue areas suggest that different nanomaterials have different effects on the structure of matrix asphalt. In addition, nano-OvPOSS influences the free volume of the matrix asphalt system most significantly. This reduces the RFV of matrix asphalt, after the addition of nano-OvPOSS secures the structural stability and the deformation resistance of matrix asphalt.

#### 3.4.3. Temperature Stability

**Figure 19:**
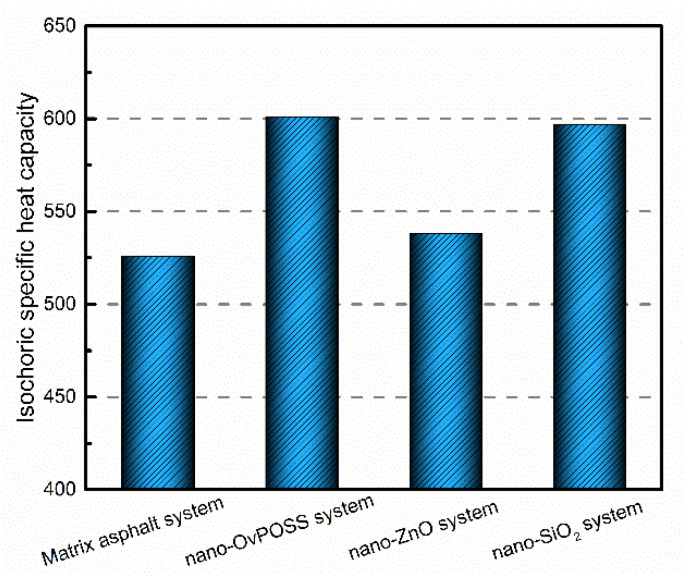
The isochoric specific heat capacity of asphalt systems.

Heat capacity is used to describe the amount of heat required to raise the temperature of a given amount of substance by 1 K. The larger the heat capacity, the more heat it absorbs when it raises 1 K. In this study, the isochoric specific heat capacity (C_v_) of the matrix asphalt system and the nanomaterial-modified asphalt systems was simulated. The simulation results of C_v_ are shown in Figure 19. It can be seen that the order of C_v_ is nano-OvPOSS-modified asphalt system > nano-SiO_2_-modified asphalt system > nano-ZnO-modified asphalt system > matrix asphalt system. Obviously, nano-OvPOSS increases the largest amount of C_v_ of matrix asphalt, which can be increased by 14.3%. Meanwhile, it is notable that the C_v_ of nano-OvPOSS-modified asphalt system is slightly higher than that of nano-SiO_2_-modified asphalt system. This is because nano-OvPOSS and nano-SiO_2_ have similar skeleton structure composed of silicon, and the silicon content of OvPOSS nanocluster is almost the same as that of SiO_2_ nanocluster. It can be seen that all of the three nanomaterials can improve the C_v_ of matrix asphalt, which suggests the addition of the nanomaterial is able to alleviate the thermodynamic instability of matrix asphalt. Significantly, nano-OvPOSS and nano-SiO_2_ perform better in improving the C_v_ of matrix asphalt than nano-ZnO.

The temperature stability is considered as an important index to measure the properties of modified asphalt since asphalt is a typical temperature sensitive material. In asphalt system, a number of molecules are gathered together through non-bond interactions. The rising temperature of asphalt system will decrease or even eliminate these non-bond interactions, thereby reducing the restraint force of asphalt molecules and diminishing the rheological properties of asphalt system. Since nano-OvPOSS-modified asphalt and nano-SiO_2_-modified asphalt are capable of absorbing more heat than matrix asphalt under equivalent environmetal conditions, these two nanomaterials will effectively slow down the rising of the temperature of asphalt system and therefore reduce the negative impact of temperature rise on the rheological properties of asphalt system.

#### 3.4.4. Resistance to Deformation

**Figure 20:**
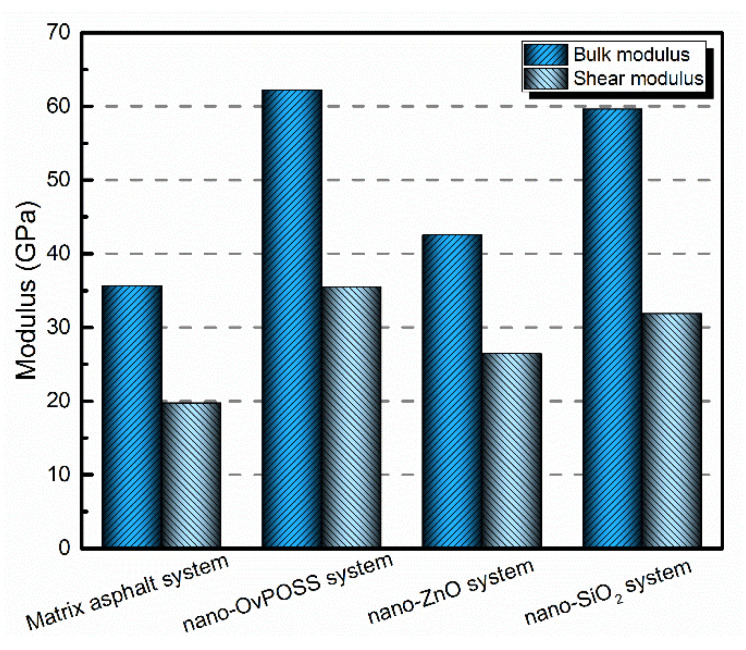
Physical moduli of asphalt systems.

To investigate the effect of the nanomaterials on the deformation resistance of matrix asphalt, the physical moduli of the matrix asphalt system and the nanomaterials-modified asphalt systems were calculated, including bulk modulus and shear modulus. The simulation results are summarized in Figure 20. As is shown in Figure 20, compared with matrix asphalt, the bulk modulus of the nano-OvPOSS-modified asphalt, nano-ZnO-modified asphalt, and nano-SiO_2_-modified asphalt increases by 74.7%, 19.4%, and 67.4%. Additionally, the shear modulus of these three nanomaterials-modified asphalts increases by 80.2%, 34.5%, and 61.9%. The increase in the modulus of each modified asphalt system shows that the three nanomaterials all have a positive effect on the mechanical properties of matrix asphalt. It is notable that the increase in the modulus of nano-OvPOSS-modified asphalt is the largest, which means nano-OvPOSS does best in improving the mechanical properties of matrix asphalt.

In general, the larger the bulk modulus and shear modulus of asphalt, the stronger the rigidity of asphalt. If the asphalt has strong rigidity, it obtains small deformation when subject to external force, which can also be defined as strong deformation resistance ability. Therefore, it can be drawn from the simulation results that the higher bulk and shear modulus of nano-OvPOSS-modified asphalt indicates that nano-OvPOSS is better than nano-ZnO and nano-SiO_2_ in improving the deformation resistance of asphalt.

Related experimental results have also proved that the mechanical properties of polymer (asphalt) can be improved with the addition of nanomaterial. Sadeghnejad et al. [54] study on the effect of nano-SiO_2_ on the mechanical properties of asphalt mixture by analyzing stiffness modulus and rut depth. They found that with the addition of nano-SiO_2_, the stiffness modulus of the asphalt mixture increases, and the rutting depth of the asphalt mixture decreases; that is to say, the mechanical properties of the asphalt mixture were improved. Turan et al. [55] investigated the mechanical properties of POSS/PLA composites (a kind of polymer system) by analyzing dynamic mechanical. They found that 3% POSS increased the Young’s modulus of POSS/PLA by 40%. Zhang et al. [56] studied the effects of ZnO on rutting resistance of the matrix asphalt and SBS-modified asphalt and found that the addition of ZnO particles can improve the anti-rutting factors of these two asphalt binders. It can be seen that the simulation results of this study are consistent with the experimental results.

## 4. Conclusions

In this paper, a comparative study was conducted to investigate the superiority of nano-OvPOSS to nano-ZnO and nano-SiO_2_, when used as an asphalt modifier by way of molecular dynamics simulation. The conclusions are as follows.

- The three nanomaterials exhibit different levels of compatibility with asphalt, among which nano-OvPOSS performs the best compatibility with asphalt, followed by nano-SiO_2_ and nano-ZnO subsequently. Nano-OvPOSS exhibits the most favorable compatibility with resinous oil out of the four asphalt components. The size of nano-OvPOSS determines its compatibility with asphalt. The smaller the particle size of nano-OvPOSS, the better its compatibility with asphalt. Therefore, of all the four sizes of nano-OvPOSS (4.4 Å, 7 Å, 10 Å, and 20 Å) adopted in this study, the 4.4 Å nano-OvPOSS exhibits the best compatibility with asphalt.
- Nano-OvPOSS is able to attract a larger and closer distribution of asphalt molecules around it than nano-SiO_2_ and nano-ZnO thanks to its distinctive characteristic of possessing eight organophilic groups, thus making it more compatible with asphalt and helping to form a more stable asphalt structure.
- The eight vinyl groups of nano-OvPOSS reinforce the interaction between molecules in the modified asphalt system, which enables nano-OvPOSS to disperse evenly in the modified asphalt system without the occurrence of aggregation. By contrast, nano-SiO_2_ and nano-ZnO, not having such special structure, are prone to aggregate in the modified asphalt system.
- The separate addition of nano-OvPOSS, nano-ZnO, and nano-SiO_2_ all leads to the shrinking of free movement space of molecules in the matrix asphalt system, yet nano-OvPOSS is able to reduce the largest percentage of the free volume of the matrix asphalt system. Such reduced RFV of matrix asphalt, caused by the addition of nano-OvPOSS, warrants the structural stability and the deformation resistance of matrix asphalt.
- Nano-OvPOSS and nano-SiO_2_ perform better in improving the C_v_ of matrix asphalt than nano-ZnO. Therefore, either the addition of nano-OvPOSS or that of nano-SiO_2_ will effectively slow down the rising of the temperature of the asphalt system and therefore reduce the negative impact of temperature rise on the rheological properties of the asphalt system.
- Nano-OvPOSS, nano-SiO_2_, and nano-ZnO all positively affect the mechanical properties of matrix asphalt, yet nano-OvPOSS results in the largest increase in the bulk modulus and shear modulus of matrix asphalt, which means nano-OvPOSS does best in improving the mechanical properties of matrix asphalt. Therefore, nano-OvPOSS-modified asphalt shows the most desirable resistance to deformation.

## Figures and Tables

**Table 1:** Parameters of the three nanomaterial-modified asphalt models.

Composition	Molecular Formula	Number
Asphaltene	C_64_H_52_S_2_	4
Resin	C_41_H_54_S	12
Saturated hydrocarbon	C_22_H_46_	28
Resinous oil	C_24_H_28_	20
Nano-OvPOSS/Nano-ZnO/Nano-SiO_2_	C_16_H_24_O_12_Si_8_/O_6_Zn_6_/H_16_O_24_Si_8_	5/6/5

**Table 2:** Information about RDF and RFV.

Type	Meaning	Equation	Explanation
RDF	The ratio of regional density to the average density.	$g\left(r\right)=\frac{dN}{\rho 4\pi r^{2}dr}$	The g(*r*) is radial distribution function; *N* is the total number of atoms; *r* is the distance; *ρ* is the density.
RFV	The percentage of volume not occupied by molecules in system.	$\mathrm{RFV}=\frac{V_{f}}{V_{f}+V_{o}}\times 100\%$	The *V_f_* is free volume and *V_o_* is the occupied volume.

**Table 3:** The distribution statistics of asphalt molecules around five OvPOSS nanoclusters.

Type	1	2	3	4	5	Average
Asphaltene	1	1	1	0	1	0.8
Resin	4	2	2	1	1	2.0
Saturated hydrocarbon	3	5	5	3	6	4.4
Resinous oil	2	2	3	3	3	2.6
Average distance/Å	12.5	10.1	11.0	10.3	11.7	11.1

**Table 4:** The distribution statistics of asphalt molecules around six ZnO nanoclusters.

Type	1	2	3	4	5	6	Average
Asphaltene	1	0	1	1	0	1	0.7
Resin	2	2	0	1	1	0	1.0
Saturated hydrocarbon	2	2	4	3	3	2	2.7
Resinous oil	5	4	2	2	3	3	3.2
Average distance/Å	14.2	15.0	13.4	14.1	13.9	14.7	14.2

**Table 5:** The distribution statistics of asphalt molecules around five SiO_2_ nanoclusters.

Type	1	2	3	4	5	Average
Asphaltene	0	1	0	1	1	0.6
Resin	2	2	2	2	1	1.8
Saturated hydrocarbon	4	1	3	4	4	3.2
Resinous oil	2	2	4	3	2	2.6
Average distance/Å	13.1	13.7	12.1	11.0	10.5	12.1

## Data Availability

The data presented in this study are available on request from the corresponding author.
